# Early prevention from the first tooth - what do German dental homepages recommend?

**DOI:** 10.1186/s12903-025-06406-3

**Published:** 2025-07-02

**Authors:** Antje Geiken, Mirja Kock, Lisa Banz, Falk Schwendicke, Christian Graetz

**Affiliations:** 1 Clinic of Conservative Dentistry and Periodontology, University of Kiel, Kiel, Germany; 2 Clinic of Conservative Dentistry and Periodontology, University of Munich, Munich, Germany

**Keywords:** Early dental screening, Dental website, Evidence-based dentistry, Internet

## Abstract

**Background:**

Preventive dental care for children should begin with the first primary tooth. New early dental screening guidelines in Germany (*Fruehuntersuchung* FU 1a-c, Engl. early dental examination [EDE 1a-c]), which were established in 2019, recommend that screening begins at six months of age. It is unclear to what extent dental practices’ websites in Germany provide such information for parents. This study aims to determine whether information on EDE is provided on German dental practices’ websites.

**Methods:**

A systematic search was conducted in three search engines between September–December 2021. To investigate their technical and functional aspects, overall quality, and the risk of bias, websites were assessed using modified validated questionnaires (i.e. LIDA, DISCERN). Demographic information and statements about EDE were examined using a range of criteria, including the presence of general information about EDE, recommendations, and other relevant content. The researchers analysed 10 websites at baseline; inter-rater reliability was 0.874 (intra-rater: 0.962).

**Results:**

A total of 80 websites were analysed. The LIDA analysis showed that 57 websites (71.25%) met at least 50% of the criteria, while the DISCERN analysis found that 78 (97.5%) met at least 50% of the criteria. EDE was mentioned on 45 websites (56.3%). Specific recommendations related to EDE were mentioned on 30 websites (37.5%), and other relevant content was covered on 15 (18.8%) websites. Further information on tooth-healthy diets was provided on 24 (30%) websites; however, only four websites (5%) included information about oral hygiene.

**Conclusions:**

While a large proportion of German dental practice websites mentioned EDE, few thoroughly explained it. Recommendations for oral hygiene and dental risk factors were also scarce. Thus, further efforts must be made to improve the provision of information on this preventive approach on the internet.

## Background

Early childhood caries (ECC) is the most common preventable childhood disease but affects more than 600 million children worldwide [1], with a prevalence of 23–90% among five-year-old youths [2]. ECC has a rapid and aggressive course [3] and can have a variety of consequences, including acute or chronic toothache, reduced food intake, abscesses, damage to tooth germs, and impaired chewing function. In addition, the treatment of ECC results in avoidable costs for the healthcare system [4].

There is a need for further research on risk factors, such as breastfeeding. For example, it is not yet clear when breastfeeding begins to have negative effects on teeth [5].

In contrast, factors at the patient level are clearer. Children from lower socioeconomic backgrounds are considered at-risk and therefore require early intervention.

Early dental prophylaxis can prevent the development of ECC and have a significant positive impact on later oral health attitudes and compliance with dental treatment [6]. Previously, a child’s first dental check-up in Germany would occur between 30 and 72 months of age. However, for children at risk of ECC, caries defects may have already developed by this time.

Parents do not seem to be sufficiently informed about critical factors related to the development of ECC, such as the consumption of fermented carbohydrates, often in the form of sweetened beverages in drinking bottles, in combination with inadequate oral hygiene [7, 8]. It is also unclear whether the information to which parents have access is fact-based.

In 2019, three new dental screening programmes (early dental examination [EDE] 1a-c, German *Fruehuntersuchung* [FU] 1a-c) were introduced in Germany. These are offered to all children with statutory health insurance—almost 90% of all children. As a part of these programmes, the first dental examination takes place by the time a child is sixth months old, after the eruption of the first milk tooth. In addition to a dental diagnosis and advice on healthy eating, an EDE includes practical advice for caregivers on oral hygiene and, depending on an individual’s caries risk, fluoridation.

This information and a referral for dental screening should be provided by a child’s treating paediatrician. Unfortunately, not all paediatricians are aware of these screenings. According to the EDE scheme, only 15.2% of paediatricians referred children to the dentist in 2020, while the majority recommended dental care for children aged 30 months and older. Worryingly, 43.2% of paediatricians considered dental checkups unnecessary for children. Thus, the dissemination of information on EDE by paediatricians remains a challenge [9]. Midwives, who are often the first medical confidants of young parents, are also not well-informed about EDE [10].

Therefore, carers often rely on alternative sources of information. The Internet is a natural resource for parents seeking information. However, there is a risk of misinformation online. On social media (e.g. Instagram, YouTube, and Twitter), subjective opinions are often spread without reflection or fact-checking [11–14]; indeed, the COVID-19 pandemic revealed the danger of misinformation on the Internet [15]. Thus, medical information should be provided on doctors’ and dentists’ practice websites. However, previous studies have found that the quality and amount of information on dentists’ websites varies [16–18].

We hypothesised that, despite the national implementation of EDE 1a–c screening programmes, German dental practice websites rarely provide comprehensive or evidence-based information about these screenings.

Therefore, the aim of this study was to determine whether German dentists’ websites contain information about EDE, focusing on (1) the technical and functional aspects of the websites, (2) the overall quality and risk of bias, and (3) the content related to dental care.

## Materials and methods

The current study was conducted according to the methodology of Geiken, Banz [19] and Geiken, Kock [16]. For further details on the methods, including the search strategy, please see our previously published investigation [16, 19].

### Sample size calculation

The sample size calculation was performed using G*Power 3.1.9.2 (University of Düsseldorf, Germany). A significance level of α = 0.05 and an effect size of 0.2 (moderate) were used to calculate the required number of sites, and the statistical power was estimated at β = 0.80. The estimate indicated that at least 64 sites should be evaluated (F = 2.52; df = 4, expected power 81%).

**Table 1:** Practice-specific parameters of the included websites

Variable and attribute	Value (*n* (%))
**Age**	
Young (up to 40 years)	10 (12%)
Middle age (41–50 years)	45 (56%)
Old (older than 50 years)	22 (28%)
No information	3 (4%)
**Practice setting**	
Single practice	35 (44%)
Multi-practice	41 (51%)
Chain practice	4 (5%)
**Specialisations**	
Paediatric dentistry	5 (6%)
Any specialisations	25 (31%)
No information	50 (63%)
**Practice location**	
Rural (fewer than 5,000 inhabitants)	5 (6%)
City (between 5,000–100,000 inhabitants)	39 (49%)
Large city (more than 100,000 inhabitants)	36 (45%)

**Table 2:** Domains regarding technical and functional aspects were assessed using a modified LIDA instrument (version 1.2)

Item	Median (IQR^a^; min–max^b^)
**2.1 Accessibility**	
• Is the information available in full text without registration, log-in, or subscription?	2 (0; 2–2)
**2.2 Usability**	
• Is there a clear statement of who this website is for?	2 (0; 1–2)
• Is the level of detail appropriate for the user’s level of knowledge? Is the layout of the main block of information clear and readable?	2 (0; 0–2)
• Is the navigation clear and well-structured?	2 (0; 0–2)
• Can you always tell your current location on the site?	2 (0; 0–2)
• Do navigational links have a consistent function?	1 (0; 0–2)
• Is the site structure (categories or organisation of pages) applied consistently?	0 (0; 0–2)
• Does the site provide an effective search function?	0 (0; 0–2)
• Can you use the site without third-party plugins?	1 (0; 0–2)
• Can the user effectively judge whether the site applies to them?	1 (0; 0–2)
• Is the website interactive?	1 (0; 0–2)
• Does the website integrate non-textual media?	0 (0; 0–2)
**2.3 Reliability**	
• Can users submit comments on specific content?	0 (0; 0–2)
• Is the site content updated at appropriate intervals?	2 (0; 1–2)
• Is it^c^ clear who runs the site?	2 (0; 0–2)
• Is it^c^ clear who pays for the site?	1 (0; 0–1)
• Can the information be checked from original sources?	0 (0; 0–2)

^a^ IQR: interquartile range

^b^ min–max: minimum–maximum

^c^ Website content

0 = never, 1 = occasionally, 2 = most of the time, 3 = always

**Table 3:** Domains regarding generic quality and risk of bias were assessed using a modified DISCERN instrument

Item	Median (IQR^a^; min–max^b^)
**3.1 Reliability**	
• Are the site’s aims clear?	2 (0; 1–2)
• Is it^c^ clear what sources of information were used to compile the publication?	0 (0; 0–2)
• Is it^c^ clear when the information used or reported in the publication was produced?	0 (0; 0–2)
• Is the website content^c^ balanced and unbiased?	2 (0; 0–2)
• Does the website provide details of additional sources of support and information?	1 (0; 0–2)
• Does the website content^c^ refer to areas of uncertainty?	0 (0; 0–1)
**3.2 Quality**	
• Does the website^c^ describe how each treatment works?	1.5 (0; 0–2)
• Does the website^c^ describe the benefits of each treatment?	1.5 (0; 0–2)
• Does the website describe the risks of each treatment?	0 (0; 0–2)
• Does the website^c^ describe what would happen if no treatment were used?	0 (0; 0–2)
• Does the website^c^ describe how the treatment choices affect overall quality of life?	1 (0; 0–2)
• Is it^c^ clear that there may be more than one possible treatment choice?	2 (0; 0–2)
• Does the website^c^ provide support for shared decision-making?	1 (0; 0–2)

^a^ IQR: interquartile range

^b^ min–max: minimum–maximum

^c^ Website content

0 = no, 1 = occasionally, 2 = most of the time, 3 = yes

**Table 4:** EDE-specific aspects; scores between 0–2 were used

Item	Median (IQR^a^; min–max^b^)
• Is early oral health screening mentioned?	2 (0; 0–2)
• Is EDE recommended?	0 (0; 0–2)
• Is EDE explained in complete sentences?	0 (0; 0–2)
• Is EDE explained in a comprehensible way?	0 (0; 0–2)
• Are the structure and classification of EDE described in more detail?	0 (0; 0–2)
• Is it mentioned that diseases and maldevelopments are recorded during EDE?	0 (0; 0–2)
• Is it mentioned that oral health behaviours are assessed during EDE?	0 (0; 0–2)
• Is the DMFT index mentioned?	0 (0; 0–2)
• Is it mentioned that EDE helps children get used to dental routines?	0 (0; 0–2)
• Is it mentioned that dental hygiene is explained in detail during EDE?	0 (0; 0–2)
• Is there any mention of nutritional counselling?	0 (0; 0–2)
• Is nutritional counselling specifically for children mentioned?	0 (0; 0–2)
• Is it explained that it is better to have few snacks between meals?	0 (0; 0–2)
• Is the ‘danger’ of bottle feeding explained?	0 (0; 0–2)
• Are hidden sugars mentioned?	0 (0; 0–2)
• Are fruit juices mentioned as a possible risk?	0 (0; 0–2)
• Is the proper timing of tooth brushing addressed?	0 (0; 0–2)
• Are hidden acids mentioned?	0 (0; 0–2)
• Is education provided about the potential risks of ignoring EDE?	0 (0; 0–2)
• Are these risks mentioned?	0 (0; 0–2)

^a^ IQR: interquartile range

^b^ min–max: minimum–maximum

0 = no information, 2 = complete information

DMFT = decayed missing filled teeth

### Functional aspects and generic risk of bias

A questionnaire was used to collect demographic data (e.g. size of city, structure of practice, age of dentists, specialisation; Table 1) and data on dental screenings (general information about EDE, recommendations and content aspects of EDE) and ECC prevention (nutrition, oral hygiene, etc.; Table 4).

In addition, the LIDA and DISCERN tools were used to analyse the websites [20, 21]. DISCERN assesses user experience, overall quality, risk of bias in health information for treatment decisions, and reliability and quality of information using an ordinal scale (0 = no, 2 = maybe, 3 = yes). LIDA captures criteria such as the accessibility, usability, and reliability of websites, with items being weighed on an ordinal scale (0 = never, 1 = occasionally, 2 = most of the time, 3 = always). The obtained scores were added to create a total score, and the percentage of the maximum possible score was calculated. Scores > 90% represented positive results, while those < 50% indicated poor results.

### Search strategy

Two independent researchers (M.K., L.B.) conducted a systematic search from 10 September–11 December 2021 using the German equivalents of the search terms ‘dentist/EDE/dental check-up’. Three search engines (Google, Bing, and Yahoo) were used for this purpose. For quality assurance, intra- and inter-rater reliability were collected. Intra-rater reliability was 0.962, and interrater reliability was 0.874. The reviewers discussed any potential discrepancies and sought the opinion of a third reviewer (A.G.) when necessary.

A total of 1,467 German-language websites based in Germany were reviewed. Of these, 1,387 were excluded based on the exclusion criteria (university hospitals, health insurance companies, health services, blogs, duplicates).

### Data extraction and analysis

The following information was obtained from the websites: demographic information (dentists age [young = younger than 41 years; middle age = 41–50 years; old = older than 50 years, and averaged for multi-practice or chain practices], as determined from CVs on the websites), specialisation (no specification, any specialisation, paediatric dentistry), practice organisation (single practice, multi-practice, or chain practice], practice name, URL, practice location (rural ≤ 5,000 inhabitants, city > 5,000–<100,000 inhabitants, large city ≥ 100,000 inhabitants), and specific German national fluoridation recommendations [22] (0 = no specification, 2 = complete specification; Tables 1, 2, 3 and 4).

### Statistical analysis

A primary statistical analysis of the data was conducted using SPSS for Mac 28.0.0.0 (IBM, Chicago, IL, USA). Statistical significance was set at *p* < 0.05. Median, quartiles, ranges, and a quality score were calculated for all categories.

Further analysis was performed using a Wilcoxon test. In addition, generalised linear modelling was used to assess the relationship between practice-related characteristics and overall quality (%). However, interaction terms were not used, as this requires additional model development and thus increases the risk of alpha inflation.

## Results

**Fig. 1:**
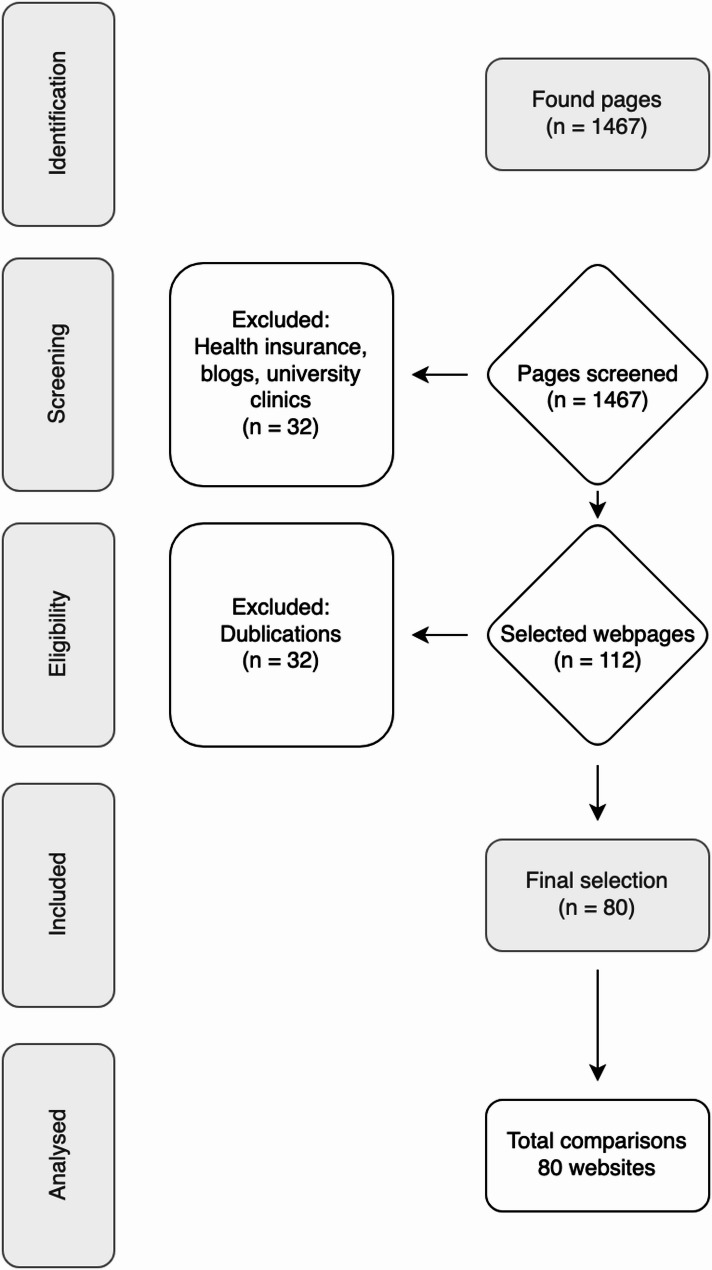
Flowchart of search and selection results

A total of 80 websites were included in the study (Fig. 1). Of these, 49% of the practices were located in cities, 45% in large cities, and 6% in rural areas. Practices with several practitioners represented the largest group (51%), followed by practices with only one practitioner (44%); only 5% were chain practices. Most websites were owned by middle-aged dentists, while those in the younger and older age ranges jointly accounted for 40% of the websites. No age could be determined for 4% of the dentists hosting the websites. No information about the dentists’ specialisations was disclosed on 63% of the websites. In total, 31% of the websites provided information on specialisations. Specialisation in paediatric dentistry was stated by 6% of the practices. These figures and percentages can be found in Table 1.

The evaluation using LIDA showed that 57 (71%) websites met at least 50% of the criteria. Most websites had adequate functionality and accessibility, were well-structured, and had easily accessible navigation options. However, there was room for improvement in the areas of interaction and searchability. In terms of reliability, it was generally clear who operated the sites. The majority of the websites were updated regularly. More than half (56%) of the websites did not list sources for the health information they provided (Table 2).

The evaluation using the DISCERN criteria showed that 78 (97.5%) websites met at least 50% of the criteria (Table 3). Although the websites conveyed information in a comprehensible manner, the sources used were not indicated. Content seemed unbiased and objective, and additional sources of information were frequently provided. The quality criteria for the information presented were partially met. Modes of action and forms of therapy were partially described; however, aspects such as uncertainty around evidence were not discussed.

Generally, there was no information on the consequences and risks of not having dental screenings or on the risks of incorrect diagnoses or over-diagnosing. A small proportion of the websites provided information on the impact of EDE on patients’ quality of life (4%) or sought to provide help in the decision-making process regarding possible therapies around ECC (5%). EDE was defined on only one (1.3%) website, but it was mentioned on 47 (58%) of the websites. Specific recommendations for EDE and caregivers were mentioned on 24 (30%) of the websites, information about the early detection of dental diseases and abnormal developments was mentioned on around 20% of the websites (*n* = 16), and diet recommendations for dental health were provided on 19 (24%) of the websites. Almost half of the websites provided information on the importance of dental hygiene (Table 4). Eight websites explained that EDE includes assessing oral health behaviour, and a similar number highlighted the risk of bottle-feeding. Only six websites (8%) advised avoiding snacks between meals. The interaction between acid levels and the timing of tooth brushing on dental health was mentioned by four (5%) websites. Regarding the wider role of acids in the diet, four websites (5%) described the influence of fruit juices, and five (6%) described the influence of hidden acids. In the multivariable analysis, no practice-related factors were significantly associated with the overall quality of the websites (Table 5).

**Table 5:** Association between practice-related factors and the overall quality score; significant associations are highlighted in bold

Factor	Beta (mean quality score)	95% Confidence Interval	*p*-value
Constant	15.3	12.8 to 18.0	< 0.001
Chained practice (ref.: multi-practice)	1.9	-0.1 to 3.8	0.062
Single practice (ref.: multi-practice)	1.5	-0.2 to 3.2	0.078
No information on specialisation (ref.: paediatric dentistry)	0.8	-0.8 to 2.5	0.324
Any specialisation (ref.: paediatric dentistry)	1.05	-0.6 to 2.7	0.201
Rural (ref.: large city)	-0.24	-1.6 to 1.14	0.737
City (ref.: large city)	-0.34	-1.0 to 0.33	0.313
Age of dentists (cont. per year)	0.003	-0.5 to 0.5	0.991
Number of dentists (cont.)	0.14	-0.16 to 0.4	0.350

rural (≤ 5,000), city (> 5,000–<100,000), large city (≥ 100,000)

## Discussion

ECC is considered a preventable disease, but it is one of the most common childhood illnesses. In Germany, dental care for children was greatly expanded in 2019. Since then, it has been recommended that children visit a dentist for the first check-up when the first milk tooth erupts. However, many parents still take their children to the dentist too late, for example after they are already in pain [23], even though studies have proven that early dental care has both medical and cost-saving benefits [24, 25].

Reasons for this include a lack of knowledge about ECC as well as a lack of knowledge about the options available. In Germany, paediatricians should provide information about EDE, and midwives are also encouraged to do so; however, this has not yet been sufficiently implemented [9, 10].

Thus, the responsibility for gathering information lies with the parents. It is logical for parents to use the Internet for this purpose, as studies show that it is increasing being used to obtain information on health-related topics. Today, 90% of the German population has access to the Internet, making dental websites an important tool for gathering information [26, 27].

The current study found that the technical and functional aspects and the structural designs of these websites were adequate. For example, there was error-free retrieval of the web pages, which was tested with the LIDA instrument [18, 28]. However, a significant limitation was that the majority of the content did not indicate which sources were used. This results reflects the findings of previous studies.

With regard to quality, which was determined using the DISCERN instrument, the websites appeared satisfactory. However, due to the specific questions of the instrument, it was not possible to assess, for example, whether the information presented stemmed from evidence-based sources or whether it reflected the opinions of the dentist or dental practice in question.

EDE was mentioned on 58% of the examined websites, and specific recommendations for caregivers were provided on 30% of the websites. However, but 1% of these websites provided a definition of EDE.

Even basic points were not explained comprehensively on all websites. For example, about half of the websites explained the importance of good oral hygiene, but few provided nutritional recommendations.

Well-informed parents are more likely to take advantage of the services offered by the EDE program. However, currently, website content does not consistently comply with national recommendations.

The lack of available information raises the question of whether this only applies to dental care for children—that is, whether this is purely a paediatric dental issue. The literature includes studies with similar research questions, for example, to examine temporomandibular joint disorders, orthodontics, periodontics, and conservative dentistry [29–31]. Akan and Dindaroglu [30] assessed the information content of dental websites on temporomandibular joint disorders and found it to be of poor quality. Another study collected data from websites of medical and non-medical authorship to investigate the fundamental topic of dentistry: caries [32]. The author rated these websites’ quality as poor, regardless of the website owner’s professional background.

The same applies to studies in the field of periodontics. Periodontitis is a disease that is becoming increasingly prevalent and is therefore a highly relevant condition; however, the topic is inadequately represented. For example, information on diagnostic procedures, treatment options, and the risks and benefits of implant treatment in a periodontally damaged jaw was missing from dental websites [31].

In summary, dental websites are not comprehensive sources of information.

This raises the question of why dentists pay so little attention to this aspect of their websites, which have a fundamental influence on patients’ treatment decisions and opinions [33].

Dentists should invest additional time and effort in maintaining and updating their websites, including writing discussions and explanations of treatment methods [34]. However, this time may not be available in their daily practice routines.

As expected, this study found no correlation between practice-related parameters, such as age, location, or specialisation. This indicates that specialist paediatric dental practices did not provide better information than general practitioners.

This study has several limitations. First, only German websites were examined, and other potential communication channels, such as social media, newsletters, or communication within the practice, were not examined, although these outlets may also influence caregiver awareness. In addition, as the content of the websites is subject to national guidelines and health policy, these results cannot be transferred to international practice. Due to the sample size, certain dental practices were not frequently represented, which may have affected the statistical power.

Furthermore, data were not collected in duplicate, but the reviewers showed good intra- and inter-rater reliability. Finally, the assessment was developed and validated by a panel of experts since there is no existing set of criteria for evaluating information on EDE.

In summary, the provision of information on the EDE on the websites of German dental practices needs to be improved.

National initiatives could be pursued to ensure a minimum level of information on EDE on websites. This would be particularly useful for countering fake news from social media [35, 36].

## Conclusions

This study examined whether the websites of German dental practices provide sufficient information on EDE 1a-c introduced in 2019. The analysis showed that although most websites met basic technical and structural quality criteria (LIDA), and many fulfilled general quality, functional and evidence-based criteria (DISCERN), there were clear deficits in the content related to EDE. While EDE was mentioned on more than half of the websites, only a minority offered detailed explanations or practical recommendations for parents. Critical aspects such as nutrition, oral hygiene, and early detection of dental risk were often missing or insufficiently addressed. Overall, the results indicate that the information about EDE on German dental websites remains inadequate, and targeted improvements are necessary to support informed decision-making by caregivers.

## Data Availability

The datasets used and/or analysed during the current study are available from the corresponding author (Antje Geiken, geiken@konspar.uni-kiel.de) on reasonable request.

## Abbreviations

ECC: Early childhood caries
EDE 1a-c (German Fruehuntersuchung = FU 1a-c): Early dental examination
DMFT: Decayed missing filled teeth
IQR: Interquartile range
min–max: minimum–maximum
